# Considering Autonomous Exploration in Healthy Environments: Reflections from an Urban Wildscape

**DOI:** 10.3390/ijerph182211867

**Published:** 2021-11-12

**Authors:** Sarah Little, Art Rice

**Affiliations:** 1 Landscape Architecture, University of Oklahoma, Norman, OK 73019, USA; 2 Landscape Architecture, North Carolina State University, Raleigh, NC 27607, USA; art_rice@ncsu.edu

**Keywords:** free-range parenting, autonomy, attachment, thick description, children and nature

## Abstract

Autonomous exploration should be considered in the creation of healthy environments since autonomy is an important developmental experience for children. For a group of boys in Raleigh, N.C., U.S. during the period 2002–2006, autonomous exploration was a meaningful experience. Results of a qualitative research project (*n* = 5) which highlight the importance of autonomous exploration are organized within a proposed framework for thick description. The framework creates verisimilitude by reporting on the context, social action and cultural context, and behavior and intentionality. The context of Raleigh and urban wildscapes furnished areas ripe for exploration. The social action and cultural context of attachment supported the autonomous exploration through scaffolded experiences of autonomy. The intentionality of the behavior was a desire to distinct themselves through a focus on individual development and the pursuit of extraordinary experiences. The ultimate outcomes of autonomous exploration for the boys were the development of long-term, intimate friendships and confidence in their decision-making ability. As cities become more health-focused, attention should be paid to preserve the rough edges of a city for children to explore.

## 1. Introduction

Frumkin et al. [1] describe healthy environments as “places in which people can grow up, live, work, play, study, pray, and age in ways that allow them to be safe and healthy, to thrive, and to reach their full potential” (p. 5). Supporting healthy lifestyles is key in creating healthy environments. In addition to clean air, water quality, and access to healthy food and green space, supporting an appropriate level of autonomy for children should be considered in the creation of healthy environments. Autonomy is a developmentally meaningful experience for children. Research suggests that children granted an appropriate level of autonomy experienced higher self-esteem, and girls, in particular, experienced lower levels of depression and higher levels of concentration than girls from families who do not grant autonomy [2]. In a study of U.S. first graders, children granted a developmentally appropriate level of autonomy experienced a lower risk of being overweight [3]. In studies from Sweden and the UK, the level of autonomy granted is associated with increased happiness in adolescents [2,4]. The developmental benefits of autonomy extend into the university years. Turner et al. [5] found that college students who were reared by parents who granted autonomy were more motivated to excel academically and displayed self-efficacy in their academic abilities.

Spatial autonomy refers to the freedom of movement and control of space that children experience as they age [6]. Spatial autonomy, primarily in the form of independent mobility, is considered in the discussion of healthy environments. Research suggests a correlation between characteristics of the built environment, such as sidewalk length, presence of street trees and green space, e.g., [7,8,9], and independent mobility. While autonomy in the built environment can take many forms from independent mobility to home range, the autonomy of interest in the article is autonomous exploration. Autonomous exploration of the physical environment happens when a child or group of children spontaneously determines the course and objectives of playful explorations of the physical environment without adult supervision or input. Autonomous exploration combines spatial autonomy with free play, i.e., activities that are freely selected and guided by the child and “undertaken for its own sake, not consciously pursued to achieve ends that are distinct from the activity itself” [10]. Two related concepts are independent mobility and home range; both concepts are spatial phenomena concerned with a distance traveled without adult supervision. Independent mobility involves a child or group of children traveling to and from a destination without adult supervision [11,12]. Home range refers to the range from a designated base or home in which a child can travel autonomously as negotiated with a parent [13,14]. While distance can be involved, autonomous exploration is not a spatial phenomenon since the character of the journey is the focus and not the distance traveled. The spontaneous and playful dimensions differentiate autonomous exploration from independent mobility and home range. While spontaneity and playfulness are not necessarily excluded from independent mobility and home range, these qualities are not inherent to the concepts as defined in the article. The spontaneity and playfulness of autonomous exploration are important dimensions as they may support higher order cognitive play behaviors, such as cooperative play and games with rules [15], as illuminated in the research presented in the article. At the heart of autonomous exploration, independent mobility, and home range is autonomy.

Although autonomy is beneficial to development, contemporary children may experience less autonomy than previous generations. Skår and Krogh [16] documented the change in a child’s autonomy over three generations in Norway. Essentially, the three generations represented were older adults, i.e., children from 1945 to 1960; adults, i.e., children from 1960 to 1980; and children. Older adults and adults reported that they directed the course of their play and no adults were nearby; whereas contemporary children were supervised more and experience less autonomy than previous generations. Skår and Krogh [16] found that adults in the study recognized the differences between their childhood and their children’s childhood experiences. Whereas the adults’ experience as children was absent of parents, their children’s experience was dominated by parents. The necessity of driving children to and from activities all but eliminated the experience of independent mobility and home range for contemporary children. Adults in the study reported considerable time spent supervising children. Where previous generations experienced autonomy in the form of a home range, the children in the study did not [16]. The trend has also been documented in the United States, e.g., [17,18,19]. The diminishing autonomy translates to children experiencing less opportunities to make decisions [20] and benefit less from autonomous exploration, independent mobility, and home range since children are chauffeured to and from destinations as seen in the United States [21], Britain [22], and Norway [16].

Since autonomy is beneficial to healthy child development, the question becomes why do children experience less forms of autonomy, such as independent mobility, home range, and autonomous exploration, than previous generations? Fear generated by stranger danger, increased traffic volumes, and cultural changes in parenting techniques are a few variables that influence the level of autonomy granted. Public service announcements in the US in the 1960s coined the term ‘stranger danger’ to warn children of the potential dangers of being abducted by strangers and had a prominent role in decreasing autonomy. The heightened awareness from ‘stranger danger’ combined with a nostalgic view of childhood where children wandered free created an environment that negatively affected the level of autonomy granted [23]. “The stranger is often invoked in cultural explanations as a symbolic, rather than real threat to children’s safety” [23]. Statistics support the belief that strangers may not be the real danger to children; most crimes perpetrated against children are committed by family members or family friends. [24,25]. In 85% of the juvenile (0–17 years old) homicides committed in the U.S. between 1980 and 2015, the offender was known, either a family member or acquaintance. Only 15% of juvenile homicides in the U.S. were committed by strangers [25]. The victim-offender relationship in the sexual assault of juveniles is no different than homicide. From 2013 to 2014, statistics show that strangers accounted for 1% of sexual assaults of children under 6 years old, 2% of the sexual assaults of children ages 6–11, and 4% of the sexual assault of children age 12–17 in the U.S. [24]. While family members or acquaintances were the primary offenders, the perceived fear generated from ‘stranger danger’ greatly impacted the level of autonomy granted to children.

In addition to ‘stranger danger’, concern surrounding traffic limits autonomy for children [26]. “It is above all unattractive living surroundings with heavy street traffic which hinder unaccompanied play and restrict opportunities for social contacts among children and adults” [27]. High traffic volumes have not always been a factor in the urban environment. Before the widespread use of automobiles, streets were a primary play area for children in Amsterdam [28]. As more people bought cars, streets were no longer safe for children to play [28]. Traffic volumes affecting children’s autonomy are not limited to the Netherlands. In the United States, children were six times more likely to play in the street at least 2 days per week when their parents perceived the neighborhood street as safe [29]. Unlike the fear generated by ‘stranger danger,’ statistics substantiate fear of traffic. In 2010, traffic crashes involving pedestrian fatalities increased almost 4% over the previous year (Department of Transportation, 2010). Of these pedestrians, 7% of fatalities and 23% of the injuries involved children ages 15 and younger (Department of Transportation, 2010). Children seem particularly vulnerable to traffic.

In addition to the threat of strangers and traffic, societal parenting trends affect children’s level of autonomy. After receiving criticism for allowing her 10 year old son to ride a New York subway alone, Skenazy [30] defended her position by coining the term free-range parenting to describe her parenting style. Free-range parents do not let fear guide their parenting decisions; instead they provide scaffolded experiences of autonomy, steer clear of over-scheduling children in structured activities in favor of unstructured play, and promote playing outside over engagement with electronics [30]. Free-range parenting harkens back to a nostalgic period in parenting where children could autonomously explore the built environment without fearing that their parents would be accused of or even arrested for child neglect and endangerment. While free-range parenting may reflect the historic role of ‘parent,’ current societal trends seem to reject this method of parenting and embrace antithetical parenting styles such as intensive mothering/parenting, excessive parenting, tiger moms, helicopter parenting, and lawnmower parenting which will be referred to as over-parenting in the article.

Societal trends embracing over-parenting styles may be attributed to the middle-class obsession in the U.S. with children obtaining admittance to top colleges and universities as seen in the phenomenon of redshirting and concerted cultivation. Redshirting is the practice of delaying kindergarten by one year [31]. While the benefits of redshirting are inconclusive [31,32,33], parents delay kindergarten entry because they believe that their child will have a competitive advantage over their peers due to the extra year of maturation [32]. Research suggests that redshirting may be tied to socio-economic status (SES). Bassok and Reardon [34] studied two nationally representative data sets in the U.S. and found that boys who are white and from high SES families were most likely to be redshirted, and schools whose populations are mostly white and have a high SES have higher rates of redshirting. The influence of SES is evident in concerted cultivation, a middle-class phenomenon meant to ensure acceptance to top universities where parents cultivate their child’s interests through extracurricular activities in order to perpetuate a middle-class lifestyle [21]. Research confirms that concerted cultivation practices are associated with parental SES [35]. In the U.S., middle-class children spent twice the amount of time in extracurricular activities than working-class children and over triple compared to children from low SES. Not only were these activities structured, but they were controlled and directed by adults [21]. Loss of free time due to participation in extracurricular activities may result in a loss of autonomy of the child and the opportunity to engage in autonomous exploration of the built environment.

In the age of tiger moms and helicopter parents, over-parenting is as associated with good parenting. The practice is so widely accepted that parents in South Carolina [36] and Florida [37] were arrested and parents in Maryland were accused of child neglect [38] for letting their children walk to and play in a park without adult supervision. Many feel that autonomous exploration should be regulated by law. According to a Reason-Rupe poll (*n* = 1000) conducted in 2014, 83% of Americans surveyed thought that laws should require supervision for 6-year-olds at a park, 68% thought that laws should require supervision for 9-year-olds at a park, and 43% thought that laws should require supervision for 12-year-olds at a park [39]. Some resolution to the situation may be achieved through legislative action as seen in the state of Utah, U.S. In 2018, Utah passed legislation protecting free-range parenting practices [40].

While the motivations behind over-parenting may not be malicious, the trend of over-parenting and denying autonomy may have negative developmental consequences. Limited autonomy in childhood and adolescence, as seen in the decrease of free play opportunities, may be correlated with the increase of mental health conditions in children, such as anxiety and depression [10]. Depriving young children and adolescents of autonomy may have consequences in emerging adulthood. In her capacity as Dean of Freshmen at Stanford University, Lythcott-Haims [20] observed parental involvement increasing so drastically that undergraduate students lacked necessary basic life skills, such as how to do laundry and deciding what to eat. Excessive parental supervision and the rejection of free-range parenting may be depriving children of important developmental experiences related to autonomy.

The reasons for decreased autonomy do not reflect malicious intent. Some of the reasons are real, some are perceived, and some reflect larger societal trends. Whatever the reasons, autonomy continues to be an experience that contributes to healthy development; however, childhood autonomy is not a ‘one size fits all’ experience. Parents and caregivers must decide the appropriate level of autonomy for their child. While age and maturity are factors in determining autonomy, the design of the built environment contributes as well. The built environment supports autonomy by supporting child-friendly modes of transportation, such as walking and bicycling; however, the built environment must inspire the child to go outdoors. The quality of the outdoor environment must motivate the child to venture outdoors and explore [41]. The inclusion of nature in urban environments seems to attract children, e.g., [42,43,44].

## 2. Materials and Methods

For generations, children have played in a creek in Raleigh, NC, U.S.; children who previously played in the creek are now allowing their children the same experience. To avoid long waits in the car rider line, adults would gather at the creek to pick children up after school. The presence of a crossing guard ensured that children safely crossed the street to meet their parents. Adults socialized with other adults or supervised play for about 30 min before leaving. Historically, play at the creek was physical, representing the functional play type. In other words, children were physically relieving the pressure of the confinement associated with being in school.

The researcher learned of the creek play from a YouTube video documenting the experience of one cohort of boys between 2002 and 2006 [45]. The video featured a photo montage of various stages of the boys’ lives from elementary school to high school set to the tune ‘Baba O’Riley’ by The Who. The photographs reflected memorable events in the lives of the cohort. Selection of photographs was purposefully choregraphed to the song lyrics. For example, a photograph displaying boys bent over digging with sticks appeared during the lyrics “I get my back into my living”. The montage highlighted **cooperative play**, e.g., selling found objects in store-fronts carved in an Elaeagnus shrub; **games with rules**, e.g., group play with rule-based affiliations; **constructive play**, e.g., creating found objects shrines in the creek bank; and **risky play**, e.g., hanging off fire escapes and rough and tumble play. Rubin [15] attributes cooperative play and games with rules as requiring a high level of cognitive skill. Playing with others requires cognitive skills in that a child must be able to control their impulses in order to play cooperatively with others, especially if their impulses are in opposition to the play objective. Games with rules involves children creating and agreeing upon the rules; cognitive skills are involved for the children to remember the rules and adjust their behavior accordingly. Risky play has been associated with positive health outcomes and increased daily physical activity and social competency [46,47]. Unfortunately, research suggests that reduced numbers of children engage in the beneficial play types featured in the video.

**Figure 1:**
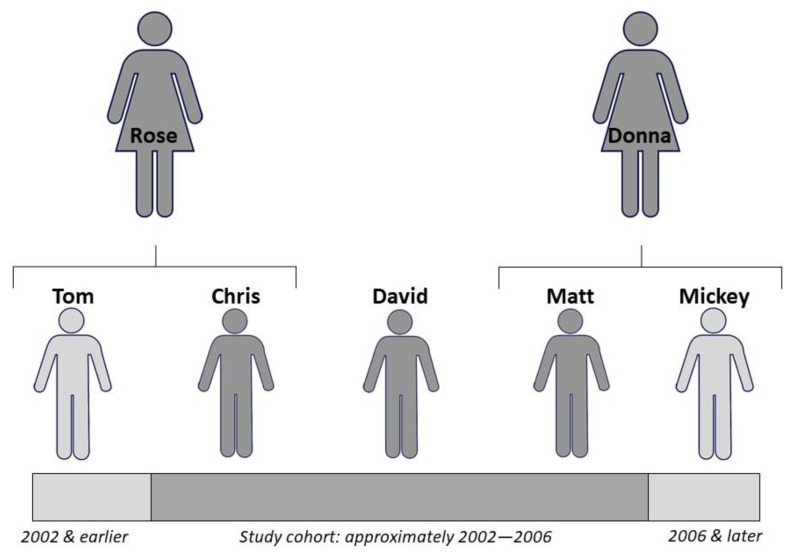
Research participants were selected to represent a timeline of the creek play to demonstrate the uniqueness of the boys’ experience of autonomous exploration [49].

At the time of viewing the video, the researcher lived two miles from the creek and tracked down the creator of the video, a mother of two of the boys featured. From this initial contact, other participants were identified and recruited through snowball or chain sampling, where initial participants identify future participants (*n* = 5; *n* = 3 boys and *n* = 2 mothers) [48]. During participant recruitment, informal interviews revealed that the research participants believed that the experience of the boys depicted in the video was unique. They described the play to be purely physical in nature at first, e.g., running, climbing, and jumping to vent the steam collected from all day in the school environment. Eventually, they developed an adult-free society with a currency system and strict rules for bartering found objects and claiming tribal affiliations which was a richness of play not obvious in the video. According to the participants, the imaginative nature of their play did not occur before or after the boys’ time at the creek. Initial casual observations of the current creek play confirmed that the tribal system play was not evident during the 2014 school year; therefore, confirming the unusual nature of the boys’ play was an important consideration in participant recruitment. Through snowball or chain sampling, the final purposeful sample represented a chronological accounting of the creek experience. One of the participants had an older brother who played in the creek after school, and another had a younger brother who played with the study cohort but could not find peers interested in playing in the creek once his brother graduated from elementary school (see Figure 1). The names of research participants were changed.

### 2.1. Research Design

Originally, the intent was to investigate the experience of the boys highlighted in the YouTube video since the high-level cognitive and risky play displayed greatly benefits child development; however, after informal interviews with participants and casual observations of the contemporary creek play, the research question began to form around understanding the contributing variables to the uniqueness of the boys’ experience. The ontological assumptions of the research favored a Constructivist-Interpretivist version of reality in that multiple versions of reality exist that are socially constructed and equally legitimate [50]. The goal of the investigation was not to launch an exhaustive probe to either prove or disprove the claims of the uniqueness of the boys’ experience; instead, the researcher’s casual observations of the contemporary creek play, and the recollections of the participants were sufficient to establish that the boys’ experience was unique.

Without fully understanding all the possible variables that contributed to the boys’ experience, a tangible research question was elusive. Instead, the data would guide the investigation; therefore, a grounded theory methodology was selected. “Grounded theory is a *general methodology* (italics in original) for developing theory that is grounded in data systematically gathered and analyzed” [51]. Collecting and analyzing data systematically in qualitative research requires special attention to the researcher since they are the instrument through which all data are analyzed and interpreted. To ensure systemic data collection and analysis, the qualitative researcher must engage in reflexivity exercises where the researcher identifies personal factors, such as socio-economic status, gender, background, values, and personal experiences. which may influence the research design and data collection, analysis, and interpretation [52,53]. Initial reflexivity exercises identify bias and an on-going reflexivity journal documenting and challenging decisions which progress the project all work together to protect against bias [52,54]. Reflexivity exercises are meant to calibrate the researcher, the main research instrument in qualitative research. In the current project, reflexivity exercises exposed the researcher’s bias toward the natural environment. Initially, the natural environment was thought to be the main variable that contributed to the boys’ unique experience; the researcher recognized this bias toward nature and challenged that assumption throughout data collection, analysis, and interpretation.

In summer 2014, semi-structured interviews occurred while the boys attended college. All methods involving human subjects received full IRB review and approval. Interviews took place at the participants’ residences (*n* = 2), workplace (*n* = 1), coffee shop (*n* = 1), or via Skype (*n* = 1). During the interviews, the researcher consulted a checklist to make sure data regarding similar phenomena were collected; however, interviews progressed like a conversation. Recorded interviews lasting between 1 and 2 h were transcribed and coded. During interviews, the mothers and boys were asked about their experiences at the creek to understand what made the experience of these boys so unique. Data were coded using Atlas.ti software (Scientific Software Development GmbH. Berlin, Germany) utilizing grounded theory coding methods which involved two main coding passes: initial and focused. The initial pass consisted of coding data line-by-line making sure to start each code with an action verb in order to “curb our tendencies to make conceptual leaps and to adopt extant theories before we have done the necessary analytic work” [52]. In the next coding pass, the focused phase, similar initial codes were grouped together and themes were identified in a process known as axial coding [52]. These themes helped to build theory, the goal of qualitative research.

Research quality was safeguarded by two standards: confirmability and transferability. The reflexivity exercises throughout the research project ensured confirmability, i.e., the quality of data and findings being confirmed [55]. Removing researcher bias from data collection, analysis, and interpretation increases the likelihood that the quality of the data and findings could be confirmed by other researchers. Transferability in qualitative research is similar to generalizability in quantitative research [55]. In qualitative research, researchers portray an account of participants’ emotion, experience, and thoughts that resonates with the reader through providing a ‘thick description’. Thick description’s goal to stimulate thick interpretation leads to thick meaning [56].

### 2.2. Thick Description

Thick description is a method of reporting ethnographic data that engages a reader’s empathy and imagination [56]. In other words, the interpretation is so ‘thick’ or complete that the description resonates with the reader. The description does not simply report the actions or behavior of the people being studied; instead, it must involve the accurate accounting of “thoughts, emotions, and web of social interaction among observed participants in their operating context” [56]. The findings resonate with readers by creating verisimilitude, “truthlike statements that produce for readers the feeling that they have experienced, or could experience, the events being described” [57].

While a definition of thick description is easily found, understanding how to produce a thick description eludes many researchers and scholars [56]. For clarity on how to create a thick description, two primary sources were consulted: Ryle [58] and Geertz [59]. Gilbert Ryle, a British philosopher, originated the term in the mid-1960s [56]. In a collection of papers from 1929 to 1968, Ryle builds the philosophical foundation for thick description by providing an example of a boy’s eye twitching [58]. The thin description of the event would be simply that the boy squeezed his eyelid. The thick description involves exploring the intentionality of the twitch. Was the twitch an involuntary eyelid movement related to a spasm, a wink conveying mischief to another, or a wink meant to make fun of another? The distinguishing variable between the examples of the boy’s eyelid twitching is intentionality. A thick description must interpret the intentionality behind the behavior.

Clifford Geertz, an American anthropologist, applied the concept of thick description to explore the influence of culture on behavior. Geertz [59] thought that “the concept of culture” … “is essentially a semiotic one” (p. 5). In Ryle’s example of the boy’s eye twitching/winking with mischief/winking as parody, the interpretation of the boy’s eyelid movements depends on the cultural knowledge of the boy’s company to decipher these signs and symbols, e.g., the intentionality of the twitch/wink. Geertz asserts that interpreting culture requires analyzing “structures of signification,” the socially established interpretations of the signs and symbols of culture [59] (p. 9). Two important variables influencing the interpretation of the cultural structures of signification are context and social action. Geertz clarifies that “…culture is not a power, something to which social events, behaviors, institutions, or processes can be causally attributed; it is a context, something within which they can be intelligibly—that is, thickly—described” [59] (p. 14). Culture is the context within which these structures of signification occur. Since the structures of signification are socially established, social action within this cultural context is important to understand. “Behavior must be attended to, and with some exactness, because it is through the flow of behavior—or, more precisely, social action—that cultural forms find articulation” [59] (p. 17).

While Geertz used thick description to report results from ethnographic studies, the research presented in the article is not an ethnography; however, the thick description as conceived by Geertz presents a nice framework to report the results of the current research. In addition to exploring the intentionality behind the behavior per Ryle, thick descriptions also should explore the structures of signification and social actions within the cultural context. Simply put, the lessons from Ryle and Geertz fit neatly within the classic questioning technique of reporters: who, what, when, where, and why. ‘When’ and ‘where’ describe the context, ‘who’ describes the social action and the cultural context, and ‘what’ and ‘why’ describe the behavior and the intentionality behind the behavior; however, results simply cannot be reported. Instead, the 5 w’s create the frame with which to focus the thick description. Attention must be given to creating verisimilitude by describing the emotion and intentionality of the behavior.

## 3. Results

Interview data are presented utilizing the proposed thick description framework of when and where (context), who (social action and cultural context), and what and why (behavior and intentionality), and findings are supported by participant quotes. From the interviews, autonomous exploration was a meaningful experience for the boys that cultivated long-term, intimate friendships and a confidence in their decision-making ability.

### 3.1. When and Where: Context

The activity under investigation occurred between 2002 and 2006 in Raleigh, N.C., U.S. North Carolina, a state along the eastern coast of the U.S., was one of the original 13 colonies and the site of the first English settlement, the ill-fated Lost Colony on Roanoke Island, North Carolina has a rich history as the birthplace of the first English child born in U.S., home to the infamous pirate Blackbeard, and site of the first manned flight by Orville and Wilber Wright. According to the 2000 Census, the population of North Carolina was 8,049,313 [60]. From 2000 to 2010, North Carolina experienced an 18.5% growth in population [61] and moved from the 11th to 10th largest state in the U.S. [62]. Much of the population expansion occurred in exurban and suburban areas around major cities [63]. The downtown areas of major cities, such as Charlotte, experienced a decline in white, non-Hispanic populations even though this demographic population increased by approximately 600,000 statewide [63]. With easy access to beautiful mountains to the west and beaches to the east, North Carolina is becoming a popular destination for retirees with white, non-Hispanic populations increasing in mountainous and beachside towns [63].

Raleigh, the City of Oaks, was established in 1792 as the capital of North Carolina and county seat of Wake County [64]. Many of the historic buildings from the settling of Raleigh remain since the city surrendered during the Civil War whereas Atlanta, GA was burned to the ground [64]. Raleigh, home to North Carolina State University, is part of a region known as the Research Triangle or simply The Triangle along with Durham, home of Duke University, and Chapel Hill, home of The University of North Carolina-Chapel Hill. A beltline (I-440), a highway that circles major metropolitan areas, contains North Carolina State University, downtown Raleigh, the boys’ neighborhoods, and the creek. The majority of the area of Raleigh inside the beltline experienced a population decline between 2000 and 2010 as more people moved to exurban and suburban areas [63].

Mordecai, the neighborhood in which the boys lived in or near is one mile north of downtown Raleigh and within a quarter mile walk to the school/creek. Once a plantation, Mordecai was subdivided in the late 1890s and early 1900s with most houses constructed between 1920 and 1940 [65]. The neighborhood was developed before automobiles were widespread in the U.S., so the scale is geared towards pedestrians instead of vehicles; therefore, the sidewalk network of the neighborhood connects seamlessly into the urban fabric of downtown Raleigh. The high walkability score of the Mordecai neighborhood garnered the title of one of the top five neighborhoods in Raleigh [66]. With the pedestrian scale and high walkability, children could travel the physical environment safely, thus supporting autonomous exploration.

In addition to the design of the physical environment, characteristics of residents attracted to living downtown adjacent neighborhoods may have supported autonomous exploration. As U.S. Census trends suggest that people were moving from inside the beltline to exurban and suburban areas between 2000 and 2010, research participants actively decided to settle in the Mordecai neighborhood. Donna fiercely rejected the notion of living in the suburbs:

*I think it is more of an attitude of not worrying and making sure that our kids have exposure to as much as we can expose them to, you know, not trying to sequester them too much in a controlled and exclusive environment, which is one reason they have always gone to public school and one reason we lived downtown. The last thing in the world we would want is them and us to be imprisoned in some sort of gated community. We are very much against that. I mean, we don’t like it. We want to meet different people.*

While peers were moving to exurban and suburban areas around Raleigh at the time, the research participants chose to live near downtown and allow their children to autonomously explore the neighborhood and downtown Raleigh.

The context of the urban wildscape was meaningful for the boys’ autonomous exploration; the presence of wildscapes inspired the boys to explore. Leftover or unoccupied spaces within developed areas have been classified as urban wildscapes, informal greenspaces, and derelict spaces. Jorgensen and Keenan [67] defined urban wildscapes as places where natural intentions are either prevailing or beginning to prevail over human intentions [53]. Urban wildscapes are not contained by scale; they can include sites as small as a crack in an impervious surface where vegetation is growing or as large as an abandoned warehouse that nature is reclaiming [67]. For the boys in the study, urban wildscapes, particularly the creek and abandoned warehouses in downtown Raleigh, were meaningful areas for play that fostered individual development.

The boys’ autonomous exploration began in the creek across the street from their elementary school. While the boys’ parents encouraged engagement with nature, some of the parents were hesitant about playing in a potentially polluted urban creek. Donna commented, *“I don’t think the creek was very clean, and so I think, in that way, maybe we were irresponsible a little bit that way.”* However, the benefits of autonomous exploration in nature ultimately outweighed any concerns over water quality.

*“I had some concern about the creek being not clean, the water, but I wanted there to be more creeks and more options since this was really the only one around. I was looking for places like the creek, and we didn’t have enough of them. The free places for kids to play, and there was very little public land, very little non-private land for the kids to play in here, and they were always being chased off, property, so I thought it was a real gift to have the creek for them to play in. They had so much fun and created these lifelong bonds that any kind of risk, health risk, I am hoping is not that great.”*

Rose felt the same as Donna regarding the benefits outweighing the risk of playing in the creek. She expressed concerns of the water quality\but understood that exposure to germs was healthy.

*“It’s funny I’m not a germaphobe and never have been. In fact, I do feel like it’s important to be exposed to more [germs] because if you’re not you’re gonna get more sick. And we’re lucky, I mean, at least as far as I knew. I hadn’t seen any major concerns. They knew not to drink it. You’ve got to be careful about that and you know if you’ve got cuts and stuff…just making sure that they were cleaned really well when they came back, but overall, I’ve always felt like it’s good.”*

Not every parent at the creek shared the opinion that the benefits outweighed the risks. Donna remembered, *“There were some parents that forbid their kids [from playing in the creek] because one parent knew more about risks of the creek sewage. So, some were banned from [playing in the creek].”*

For the boys, the creek provided an escape from adult restrictions. Matt described his motivation in going to the creek:

*I think a lot of it was wanting to be unsupervised and have the freedom to like do whatever you want really and kind of go wild, because after being in school all day and having teachers telling you: you can’t go behind the trees, you can’t go in like certain corners of the playground. You just really are ready to learn for yourself and have some freedom and not have someone tell you what to do all the time.*

The creek was the boys’ retreat from adult control in both physical qualities and the amount of autonomy experienced. The wild, overgrown characteristics of the creek were in sharp contrast to the orderly, adult-controlled environment of school. At school, environmental qualities were used to restrict movement, e.g., “*you can’t go behind the trees, you can’t go in like certain corners of the playground”.* At the creek, environmental qualities spurred imagination and play. David explained, *“You’ll find a really deep section of the creek or something. Like wow that’s really neat or like some big vines and I just remember feeling like I was in Vietnam or something like on some like expedition.”* David continues, *“I think it was just kind of like a new… kind of like a new frontier almost.”*

Without the presence of adult control at the creek, the boys enjoyed a high level of autonomy which greatly influenced their play behavior and the creation of intimate, long-lasting friendships. David recognized,

*And I feel like the reason why we were hanging out with each other was because we had parents that were, you know ‘ok yeah you guys can do that’ …and the parents that were like ‘uh we want you to stay inside the house’ or you know ‘stay within this block’ then we weren’t really hanging out with them because we were outside the block.*

Caregivers were excited by the boys’ creek play and supported the activity. Rose explained, “*There really wasn’t much close supervision at all, and we found that they usually did better if there wasn’t. Because they were much more creative. They knew what they were doing.”*

The creek became the boys’ network to explore other urban wildscapes of Raleigh. Chris recalled,

*The creek was our place to explore. It was our little realm where we could go and see everything there was to see and as we got older and we all got bikes and our parents let us roam around*

*Raleigh as opposed to just the creek. Raleigh—I would say became our creek in a way because it was just our place to explore.*

Eventually, the creek became too small. David explained, *“The creek got too small. Then like you know we had to go the neighborhood and then the neighborhood got too small. Then we had to go like you know all of downtown Raleigh.”* The Warehouse District in downtown Raleigh became a another meaningful urban wildscape. At that time, the district was filled with massive, abandoned buildings ripe for exploration. The boys recalled stories of exploring and climbing to the rooftops of the abandoned buildings. David remarked on the coffee shop where the interview took place, *“And even this, for instance, used to be, before they redid it, it was just like an abandon building that had like all this stuff and you could get on top of the roof really easily.”* Unfortunately, most of the urban wildscapes have disappeared from the Warehouse District. While the downtown area of Raleigh was experiencing a population decline between 2000 and 2010, that would change when CAM Raleigh, a contemporary art museum, and Citrix Systems Inc., a tech giant, moved into the warehouse district in 2011 and 2014 respectively. The addition of these entities in the Warehouse District spawned renewed interested in the area and eventually renovation. The boys’ time of autonomous exploration of urban wildscapes represents a unique space within the timeline of development in downtown Raleigh, N.C.

### 3.2. Who: The Social Action and Cultural Context

The social action of the boys expressed in the interviews was enchanting; the parents and boys fondly recalled magical adventures of autonomous exploration. At first, the play was purely physical; running, jumping, and climbing trees to release the energy built up from a day of being at school. As the boys became more familiar with each other, the play evolved into a rich, complex society with rules segregated from adults. Matt described, *“That was more of the evolution because as we all kind of got to know each other. That was like the product of getting to know each other and playing with each other.”* The imaginative society was complete with group associations. The Munchkins group was for the shorter boys, and the Tree group was for older boys who congregated in the trees growing along the creek banks. There were also the Monkeys and the Pirates. While the society had separate groups, the boys still played together. Chris explained, “*It was never like we wouldn’t like entirely separate ourselves but there was kind of like you know okay that the munchkins are doing this and the tree tribe is doing this.”* The parents delighted in hearing stories of their autonomous exploration at the creek. Donna recalled, *“They would have, like, little wars, and they took it really seriously. They would steal from each other, and, you know, it was like a little caveman society.”*

One social act in which everyone engaged was commerce. The boys developed not only currency but rules for bartering. Rubin [15] defines to this type of play as ‘games with rules’ where a group’s behavior conforms to an agreed upon set of rules which represents the highest form of cognitive play. Matt described the commerce system, “*We all have different shops where we would sell little pieces of glass.”* The boys collected objects that washed up in the creek and traded them for other objects. An overgrown Elaeagnus shrub growing along the banks served as ‘The Shops,’ storefronts in which the bartering activities occurred. The boys carved dens into the dense Elaeagnus and created storefronts where they traded found objects. Chris explains his creation of ‘Creek Currency’ to enhance the bartering system, *“I designed like some money that I called Creek Currency on Word software and printed them out. It kind of looks like a dollar and it said, ‘Creek Currency’ on it, and they were all one dollar Creek Currencies.”*

The boys also celebrated creek Christmas in ‘The Shops’ area. They collected discarded Christmas trees from the curb, dragged them to the creek, and displayed them in their storefront. Donna explained, “*They would steal discarded Christmas trees from the street and bring them down there [the creek] and then give each other creek gifts, and then they would have, like, little wars, and they would steal each other’s trees.”* Some discarded conifers came with the silvery Christmas tinsel that the boys enhanced with their found objects. As the festivities of Creek Christmas wound down, the boys exchanged gifts of precious found objects collected over the course of the year. ‘The Shops’ area became central to their play and the social action supported by the creek.

Examining the social actions of other children and caregivers within the neighborhood associated with the elementary school during this time presents an interesting contrast to the study cohort. Not every adult in the neighborhood was enchanted by the boys’ autonomous exploration; some mirrored the societal trend to reject a free-range parenting style as explained in the Introduction. Donna was ostracized among other caregivers for granting autonomy.

*“There were some parents who were trying to tell the kids to put the sticks down and “Let’s play nice” and “You can’t do that,” and it was just way too much parental interference [at the creek]. There sometimes will be accidents, but my experience has been there has never been any great harm done to anyone. I would much rather my kids grow up… having a full experience of life and having some adventure than, just being chauffeured around in air-conditioned minivans to their next lesson or sports event. To me, that is not a childhood.”*

Conflicts among caregivers extended beyond the creek. Donna remembers,

*“There was a neighbor. She had an only child, very protective of him. I guess the school was having a fair, and I offered to watch her child and another neighbor’s child. They’re both very protective, and they wouldn’t let me. They would not leave their kids with me because I was too cavalier, and I was not protective enough of children. So, yeah, there is some backlash with parents who, in my mind, are hung up and worried too much, and that is their issue.”*

Regardless of the negative reactions that the boys’ autonomous exploration solicited from other caregivers, the boys’ parents continued to grant autonomy.

The cultural context of the autonomous exploration of the boys is understood best through the lens of human attachment. Attachment refers to the relationship between a child and a caregiver. In secure attachment, the child knows that they can depend on the caregiver for protection; the caregiver is responsive and consoling [68]. The caregiver becomes a secure base in that the securely attached child ventures from the base/caregiver but returns when stressed for comfort [69]. In secure attachment relationships, a pattern is established by the movement of a securely attached child orbiting around the secure base. The interview data revealed that the boys were securely attached to their parents. While autonomously exploring, the boys managed risks which they retold to their parents, who represented a secure base. David recalled an encounter with a suspected rabid raccoon while walking along the creek, *“One time, a raccoon that obviously had rabies was like, trying to get us. I always remember, running just so far away, but yeah I mean, that sort of thing, I guess makes your courage go up*.” Matt describes a scary encounter with a man who tried to steal his bike, “*Wow it would be really bad if he caught me, but I am on a bike so I can probably get out of here before he gets me.”* The boys also encountered unhoused people camping along the creek. Donna confirms, *“They would come across homeless people camping.”*

The caregivers in the study acting as the secure base tolerated the ‘risks’ the boys encountered because they had similar adventures. As children, Donna and Rose autonomously explored nearby nature and wildscapes. They recognized the meaningfulness of autonomous exploration to their individual development and wanted their children to have similar experiences. Both Rose and Donna played in creeks as children. Rose recalled, *“I grew up here in Raleigh and back behind our house we had a creek, and we use to play back there all the time.”* Donna remembered, “*There was a creek at the bottom of our street, and we used to go down there and play and walk in the woods, and in the winter, it iced over, and we would go ice skating.”* Donna also recalled playing in wildscapes, *“There was also an old, abandoned stone estate that was nearby, and we used to go there and make Dracula movies.”* Rose and Donna’s respective parents were decidedly absent from these adventures of autonomous exploration. Rose talked about coming home from the creek and reporting her adventures to her mother, and Donna recalled roaming around town without adult supervision, *“Never, ever* [would there be adult supervision]*, and, I mean, we would walk all over the town. I would go downtown to movies, and I would go out for lunch by myself from second grade on.”* The caregivers told the boys stories of their autonomous exploration. Chris recalled stories his parents shared of their nature play. *“I think that when my mom was young, she had woods in her backyard and so did my dad you know, and they would go, and they would play in the woods.”*

Within the secure attachment relationship, the original social action that afforded the boys’ autonomous exploration at the creek was the action of the caregivers who scaffolded opportunities for autonomy by providing age-appropriate experiences which supported a high level of autonomy later. Rose recalled, *“When he was little, it was just me making sure that he was safe, but as they got older the creek’s not very deep, so there really wouldn’t be an issue with that* [no supervision]*.”* Rose provided early scaffolding experiences for Chris while his older brother, Tom, played with friends in the creek after school. Chris as a toddler would run to the creek, splash around, and run back to Rose, thus mimicking the secure base orbiting pattern of secure attachment relationships. While Rose was always close, the distance between Rose and Chris as a toddler gave him the sense that he was exploring the world alone. Not only did Rose provide a scaffolded experience of the creek, she also provided a scaffolded experience of autonomy. Chris explained, *“I can remember when I was younger like before the creek days that my parents would take me to playgrounds and they would be there, but they were never follow me around. There was always a certain level of freedom.”* Chris’ early exposure to autonomous exploration would prove to be a meaningful experience as he would eventually lead Matt to play in the creek after school. Matt explained, *“[Chris] was one of the first friends I met at Conn. So, I would just start to follow him down [to the creek].”* Eventually, the other boys joined. Chris’ early scaffolded experience of autonomy at the creek served as the catalyst for the uniqueness of the boys’ autonomous exploration and the creation of the adult-free society.

The cultural context of human attachment may have been expanded to include the physical environment. The pattern of a child orbiting around a secure base was seen in the boys’ relationship with their caregivers. They managed risks during their autonomous explorations and shared stories of these experiences with caregivers. From the interview data, the orbiting pattern was observed in the boys’ movement around the creek. No matter where their autonomous explorations lead, the boys always found their way back to the creek. While the main creek activity occurred during the boys’ elementary school years (Kindergarten through 5th grade), the boys frequently returned. David confirmed, *“We still hung out, and periodically, we would meet up down there [at the creek]. We would climb trees and stuff. And so that went on until probably like most of 7th grade.”* In high school, Matt wanted to share his childhood experiences at the creek with his new friends. Matt explained,

*“A lot of times it was kind of like trying to show [high school friends] our lifestyle to get them to understand and like it is funny like bringing people to the creek and showing them around Raleigh and like ‘That is what we used to do.’”*

**Figure 2:**
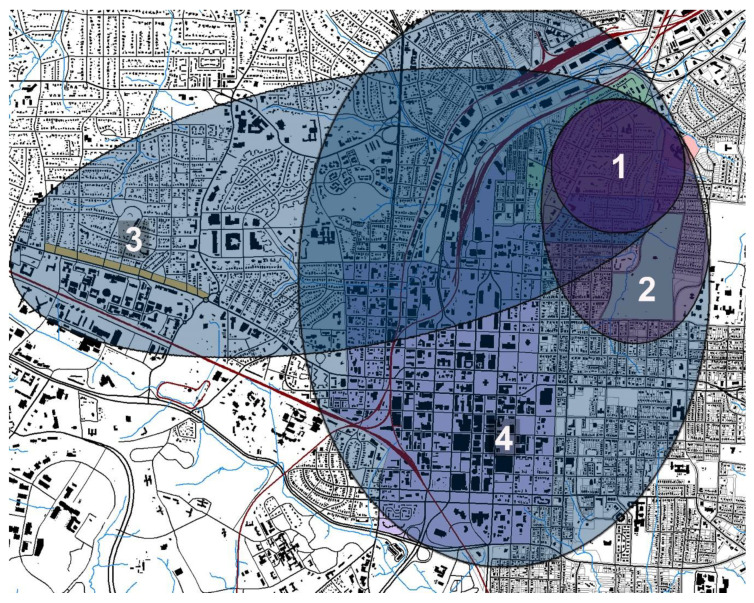
The creek, elementary school, and most of the boys’ homes appear in Zone 1, approximately half a mile radius. From the creek, the boys ventured to Oakwood Cemetery [44].

Little and Derr [44] suggest that the secure base in human attachment mirrors home range, i.e., the distance from a designated base or home which a child can travel autonomously as negotiated with a caregiver [13,14]. As the boys grew older, their autonomous explorations and home range expanded beyond the area of the creek (see Figure 2). Chris explained,

*The creek was our place to explore, and it was like our little realm where we could go and see everything there was to see and as we got older, and we all got bikes and our parents let us roam around Raleigh as opposed to just the creek. Raleigh—I would say became our creek in a way because it just was our place to explore.*

Just like the caregiver in the secure attachment relationship, the creek provided safety and protection for the boys. It was the spine of the neighborhood which safely connected the boys to other areas of Raleigh, N.C. It was part of a riparian network that provided safe passage for the boys to autonomously explore the city beyond the creek. Matt explains, *“Sometimes it was just about exploring the creek. Like seeing how far up the creek we would go, and where we haven’t been before and just kind of figuring out where the creek went and figuring out our neighborhood.”* While the riparian network was separate from vehicular traffic, there were still potential risks that the boys encountered. As mentioned earlier, the boys managed environmental risks, such as the encounter with the suspected rabid raccoon and navigating the wild, overgrown landscape of the creek, and social risks, such as the unhoused camping along the creek. From this exposure, the boys understood how to navigate risks. For example, the boys understood how to approach unhoused people and were not intimidated, an experience which distinguished them from peers. David observed, *“Growing up in this area you know how to handle homeless people coming up. Everyone else is like getting scared and stuff and we can have conversations with them and like you know be friendly with them and stuff.”*

Eventually, the boys began to autonomously explore areas of Raleigh away from the creek. They soon discovered that the railroad tracks formed a network connecting them to Raleigh like the riparian network of the creek. The tracks provided an environment for the boys to continue navigating risks; however, their experiences at the tracks were informed by their experiences at the creek. Like their parents scaffolding autonomy for the boys when they were younger at the creek, the range of the boys’ environmental experiences were scaffolded as well. David explains, *“Climbing on trains is kind of fun, and it’s kind of like climbing a tree.”* He continues, *“They have stuff like a bunch of piles of junk and stuff. We kind of sit on it, and it’s kind of like being under some bushes.”* The early experiences at the creek informed future experiences of autonomous exploration.

The tracks eventually led the boys to downtown Raleigh and the Warehouse District which at the time was an urban wildscape filled with abandoned warehouses ready for autonomous exploration. David recalls,

*We climbed a lot of the cranes downtown. Then we climbed a lot of the buildings. There’s one warehouse that was where the new Citric’s building is. You can get on top of it pretty easily. It had a really nice view, and so we hung out there like a whole lot of nights or like a lot of mornings I guess we’d go there and watch the sunrise.*

Through the social action and cultural context of the boys’ experience, the need to consider autonomy in the creation of healthy environments is illuminated through the developmental outcomes the boys experienced. The social actions demonstrate play that requires higher order cognitive skills than purely physical play. These imaginative endeavors fostered the creation of intimate, long-lasting friendships. Although the boys went to different middle and high schools and eventually different universities, they remained close, intimate friends. Matt explains,

*I think about my friends, like my neighborhood friends, that I grew up with. We will just like never go away. Like it’s not an option. They are close as family to me. Whether we get into a fight, and someone stomps out of the room, it is not even a question like they are going to come back 20 min and it is going to be the same.*

Chris confirms, *“Those friendships were like almost separate in the world or like in my other friendships they were kind of different. It was just a very different thing, and I don’t think I have ever made other friendships like that.”*

The strength and longevity of their friendship stem from their shared experiences of autonomous exploration and navigating risks. Matt explained,

*I definitely think of them differently just because of the different things that we have been through together and like the different obstacles we have been through together. Just like really great and shitty times together that we experienced in downtown but yea it has been phenomenal.*

Rose confirms,

*All of the sudden here’s just this perfect environment for them to develop as friends, develop as individuals, to learn so much, to learn how to interact with each other, to learn how to have fights, to learn how to solve you know to resolve those issues. I think it would have been very different [without the creek]. I mean I still think they probably would have been friends, but I think their paths may have gone different ways. It’s an amazing impact that this one place had on them.*

The cultural context of human attachment highlights how the boys were prepared for future autonomous exploration from early scaffolded experiences provided by parents at the creek. These social experiences of scaffolding translated into environmental experiences of scaffolding as the boys explored more developed areas of Raleigh. Through their autonomous explorations, the boys navigated environmental risks, such as encounters with wildlife and climbing trees and later cranes and buildings in Downtown Raleigh. Their successful management of these risks became sources of pride and built confidence in their ability to navigate risk in emerging adulthood. At the time of the interviews, the boys were college students and had just returned from a trip to Peru. The boys interviewed expressed pride in this trip in that they went without the safety of a tour group. David explains, “*A lot of my friends at App, for instance, are going out of country, but like everyone that was going out of country was like going through like a program or something.”* Like the risks the boys managed at the creek and Downtown Raleigh, the boys had the opportunity to manage risks in their adventures in emerging adulthood. David recalled, *“We got in, you know, funny situations. We were always able just to like ‘haha, like, we’ll get through it.’ I mean we always did too. That upbringing made us more comfortable in that situation.”* Their sense of adventure and willingness to take risks were cultivated through years of autonomous exploration as children. Matt explained*, “Just us as a group being able to judge like how bad certain places are and how we need to be careful. Because there would be so many times downtown we shouldn’t go there because that looks like the ghetto. We shouldn’t go hang out over there. Just being able to mature your judgment.”*

### 3.3. What and Why: Behavior and Intentionality

The desire to distinguish themselves was a motivating force for the families that participated in the study. Distinction came from a focus on individual growth and the pursuit of extraordinary experiences. As discussed in the Introduction, concerted cultivation is a middle-class parenting trend whose aim is to socialize their children to obtain a middle-class existence through importance of getting accepted into a good university through scheduling them in extra-curricular activities. Parents interviewed for the study focused on the individual growth of their children instead of conforming to concerted cultivation. Instead of enrolling their children in extra-curricular, structured, adult-led activities, study parents allowed children to select their own after-school activities. Rose approached parenting from the standpoint of helping her children discover what they loved. She observed that her oldest son, Tom, preferred structured, science-focused programs, and Chris preferred limited structure. Therefore, Rose enrolled Tom in classes at the Natural Sciences Museum and allowed Chris to autonomously explore after school. Rose explains, *“My husband and I both have been strong believers in that and starting from a very early age to you know to go and explore and find out what makes you happy and what makes you tick.”*

Donna was motivated by the pursuit of extraordinary experiences. She explains,

*We have always been adventurous, going places and visiting other cultures. My husband and I met in the Peace Corps in Africa, and in fact, we are headed back to Africa next week with the whole family. And I think being open to diversity and other cultures and other people has always been something we have supported and been interested in.*

The boys embraced the pursuit of extraordinary experiences. They created these experiences at the creek and downtown Raleigh as boys and teenagers respectively. As emerging adults, they planned adventure-based trips together during summer vacations from university, such as hiking the John Muir Trail in California or touring around Peru without the safety of a tour group. At the heart of these extraordinary adventures was the scaffolded autonomy the boys experienced as children. Through a series of scaffolded, age-appropriate opportunities of autonomy, the boys managed risks and developed a confidence in their decision-making ability while developing long-term friendships.

## 4. Conclusions

Autonomy, an experience that supports healthy child development, is considered in the creation of healthy environments in the form of independent mobility. While independent travel is an important experience for children, autonomy in the built environment should also include autonomous exploration, i.e., when a child or group of children spontaneously determines the course and objectives of playful explorations of the physical environment without adult supervision or input. Autonomous exploration may facilitate higher order play behavior, such as cooperative/social play, dramatic play, and games with rules [15] and therefore, warrants attention.

Utilizing the classic questioning technique of who, what, when, where, and why, a framework for thick description is proposed to highlight the experience of a group boys playing in Raleigh, NC from 2002 to 2006 and the importance of autonomous exploration. When and where describe the temporal and physical contexts, and who describes the social action and cultural context since Geertz [59] suggests that behavior is influenced by social action and culture and must be situated within a context. What and why describe the behavior and intentionality which Ryle [58] identifies as important components of a thick description.

The thick description highlights the many factors which combined to make the experience of the boys possible. Urban wildscapes within Raleigh facilitated the autonomous exploration. The creek was left undisturbed, wild, and natural; abandoned warehouses in downtown Raleigh were vacant voids ripe for exploration with the reality of renovation a few years away. The absence of adults at the creek facilitated the creation of the boys’ imaginative society complete with rules of conduct and commerce. The autonomous explorations at the creek morphed into explorations of the railroad tracks and downtown Raleigh as the boys aged. These left-over, rough edges of the cityscape became magical places for the boys to develop. As attention to healthy environments continues to grow, attention must be paid to preserving the rough edges of a city for children to explore.

## Data Availability

Data are not available as the study participants did not grant permission to make raw data available.
